# Supplementary material for: Complete Genome Sequencing of Tick-Borne Encephalitis Virus Directly from Clinical Samples: Comparison of Shotgun Metagenomic and Targeted Amplicon-Based Sequencing
Source: Viruses. 2022 Jun 10;14(6):1267. doi: 10.3390/v14061267 (PMC9231111; doi:10.3390/v14061267)
Supplement: Supplementary file 1 [file viruses-14-01267-s001.zip › Supplemental tables/Supplemental Table S1.pdf]

**Table S1: Table with newly designed primers pairs, sequences size and pools**

| Primer name | Pool | Sequence | Size | % GC | Tm |
|---|---|---|---|---|---|
| TBE_LJ_1_LEFT | 1 | TAGCAGCGGTTGGTTTGAAAGA | 22 | 45.45 | 60.93 |
| TBE_LJ_1_RIGHT | 1 | CTGACCTCCTTTTCCCACGTTT | 22 | 50.00 | 60.93 |
| TBE_LJ_2_LEFT | 2 | CGTTCTGGAACTCAGTCCCTCT | 22 | 54.55 | 61.59 |
| TBE_LJ_2_RIGHT | 2 | GTTTCCCACAGCGTCCATACTC | 22 | 54.55 | 61.43 |
| TBE_LJ_3_LEFT | 1 | AGATCAAGGAGAAGAGCCTGTTG | 23 | 47.83 | 60.31 |
| TBE_LJ_3_RIGHT | 1 | TCCCCTGAGTACCAGTCACAAA | 22 | 50.00 | 60.88 |
| TBE_LJ_4_LEFT | 2 | TTGTGTGGTTGACCCTGGAGAG | 22 | 54.55 | 62.32 |
| TBE_LJ_4_RIGHT | 2 | TCCAAACAGTCCACAGTGGTTG | 22 | 50.00 | 61.13 |
| TBE_LJ_5_LEFT | 1 | CACTTTGGCTGAGGAACACCAG | 22 | 54.55 | 61.83 |
| TBE_LJ_5_RIGHT | 1 | TGTTCCACTGTCTTGTCAAGCT | 22 | 45.45 | 60.28 |
| TBE_LJ_6_LEFT | 2 | GGAGATGTGTCCTTGTTGTGCA | 22 | 50.00 | 61.25 |
| TBE_LJ_6_RIGHT | 2 | GCTCTCTTCCATGTGAACTTTGTT | 24 | 41.67 | 60.04 |
| TBE_LJ_7_LEFT | 1 | CTGCGAAGTGGGACTGGAAAAA | 22 | 50.00 | 61.51 |
| TBE_LJ_7_RIGHT | 1 | CGTGCTCTCCTATCACTGTCAG | 22 | 54.55 | 60.4 |
| TBE_LJ_8_LEFT | 2 | GTTCCAAAAAGGGAGCAGCATC | 22 | 50.00 | 60.53 |
| TBE_LJ_8_RIGHT | 2 | TTCTGAGACCTCTCTCCACACG | 22 | 54.55 | 61.32 |
| TBE_LJ_9_LEFT | 1 | ATGAGCTTCCTCTTGGCTGGAG | 22 | 54.55 | 62.26 |
| TBE_LJ_9_RIGHT | 1 | CCAGGAGACTTTTATGTCCTTTCCT | 25 | 44.00 | 60.67 |
| TBE_LJ_10_LEFT | 2 | CAGTGGTGGTGGACAAGTTTGA | 22 | 50.00 | 61.13 |
| TBE_LJ_10_RIGHT | 2 | GTCAGTGACCAAAAGTTCAACTATGT | 26 | 38.46 | 60.4 |
| TBE_LJ_11_LEFT | 1 | ACATGAGTGTGACACAGGAGTG | 22 | 50.00 | 60.4 |
| TBE_LJ_11_RIGHT | 1 | TCTGGGATAACCTTGCCACTCT | 22 | 50.00 | 61.02 |
| TBE_LJ_12_LEFT | 2 | CCGAGTCATCAGAGAGGAGTGT | 22 | 54.55 | 61.13 |
| TBE_LJ_12_RIGHT | 2 | ACATAGCGCACCAGACTCTCTA | 22 | 50.00 | 60.87 |
| TBE_LJ_13_LEFT | 1 | GTGGCATTGTTTGTGGTCCTTG | 22 | 50.00 | 60.98 |
| TBE_LJ_13_RIGHT | 1 | CCGTGCAAGCCCTGAATATCAG | 22 | 54.55 | 61.82 |
| TBE_LJ_14_LEFT | 2 | TTTTGCTGGTCCTGGAATTGGG | 22 | 50.00 | 61.53 |
| TBE_LJ_14_RIGHT | 2 | ACTGGCCAGTGTTAACATGACC | 22 | 50.00 | 61.00 |
| TBE_LJ_15_LEFT | 1 | CTGTCAGCTCACAGAGGAAGAC | 22 | 54.55 | 60.53 |
| TBE_LJ_15_RIGHT | 1 | CACAGTCCCATCACACCAAGAA | 22 | 50.00 | 60.67 |
| TBE_LJ_16_LEFT | 2 | AATGATGGCTTTTTGGCTGCTG | 22 | 45.45 | 60.8 |
| TBE_LJ_16_RIGHT | 2 | TTTTTCCTCCAGACTCCAAGCT | 22 | 45.45 | 59.74 |
| TBE_LJ_17_LEFT | 1 | GTCTATTGATGACGCTGTGGCC | 22 | 54.55 | 61.8 |
| TBE_LJ_17_RIGHT | 1 | GATCTGACCCTTTGCTGTCCAG | 22 | 54.55 | 61.12 |
| TBE_LJ_18_LEFT | 2 | GAGACCTACGTCAGCAGCATTG | 22 | 54.55 | 61.48 |
| TBE_LJ_18_RIGHT | 2 | CCAATGGGCCTCATCCATGATT | 22 | 50.00 | 60.94 |
| TBE_LJ_19_LEFT | 1 | CAACCTATGTCAACAGACGGCT | 22 | 50.00 | 60.79 |
| TBE_LJ_19_RIGHT | 1 | CCTTTTCAAAGGTTTTGCTGTTCAA | 25 | 36.00 | 59.97 |
| TBE_LJ_20_LEFT | 2 | CAAAGGGTGGTGCTATAGCTCG | 22 | 54.55 | 61.24 |
| TBE_LJ_20_RIGHT | 2 | CGCAAGGTTGTTATGTTGTCAAGA | 24 | 41.67 | 60.58 |
| TBE_LJ_21_LEFT | 1 | TGGACAGTGTGATGATGATGACA | 23 | 43.48 | 59.99 |
| TBE_LJ_21_RIGHT | 1 | TCACGTCCCTCTTTGAACATGC | 22 | 50.00 | 61.31 |
| TBE_LJ_22_LEFT | 2 | GATCGAAGCTGGACATGGGAAG | 22 | 54.55 | 61.24 |
| TBE_LJ_22_RIGHT | 2 | GACGACGAAACACCAGATGACA | 22 | 50.00 | 61.04 |
| TBE_LJ_23_LEFT | 1 | CCCCAGAGGCCTTTCTGACTAT | 22 | 54.55 | 61.42 |
| TBE_LJ_23_RIGHT | 1 | ATTCGTCCATTCACTCCATGGC | 22 | 50.00 | 61.19 |
| TBE_LJ_24_LEFT | 2 | TTCTGGAGAAGACCAAGGCAGA | 22 | 50.00 | 61.21 |
| TBE_LJ_24_RIGHT | 2 | AGACCTTGTGAGCTCTCTGTGT | 22 | 50.00 | 61.2 |
| TBE_LJ_25_LEFT | 1 | CCAACAACTTGTCAACAGTGCC | 22 | 50.00 | 60.91 |
| TBE_LJ_25_RIGHT | 1 | CTGTTATGGAGGCCACTGTTCG | 22 | 54.55 | 61.43 |
| TBE_LJ_26_LEFT | 2 | AGGAAAATGAGTCTGGTGTTAGCC | 24 | 45.83 | 61.13 |
| TBE_LJ_26_RIGHT | 2 | TTGGTCTCTCCTCTTCTGAGCA | 22 | 50.00 | 60.68 |
| TBE_LJ_27_LEFT | 1 | CACCAGGGAGGAATTCTTCGTG | 22 | 54.55 | 61.12 |
| TBE_LJ_27_RIGHT | 1 | GCTCTCTCCGATGTCACACATG | 22 | 54.55 | 61.23 |
| TBE_LJ_28_LEFT | 2 | CAAGCCTGGGTTGGAACTTGAT | 22 | 50.00 | 61.27 |
| TBE_LJ_28_RIGHT | 2 | CTGGTCTCCAAACCGAGCTAAG | 22 | 54.55 | 60.85 |

| | | | | | |
|---|---|---|---|---|---|
| TBE_LJ_29_LEFT | 1 | TACTCAACAGCTGTCACTGGGA | 22 | 50.00 | 61.2 |
| TBE_LJ_29_RIGHT | 1 | TTTGAACACTCTCTGCTGTCCG | 22 | 50.00 | 60.98 |
| TBE_LJ_30_LEFT | 2 | AACTTCTCAGCTGGCCATGGAA | 22 | 50.00 | 62.41 |
| TBE_LJ_30_RIGHT | 2 | CCCAGTTTCTTCTCTCTCTTGCC | 23 | 52.17 | 61.12 |
| TBE_LJ_31_LEFT | 1 | GAGAGAAAGGCACCTTATGGGG | 22 | 54.55 | 60.61 |
| TBE_LJ_31_RIGHT | 1 | TGGTTGCCAATTGTTTGTGCTC | 22 | 45.45 | 60.92 |
| TBE_LJ_32_LEFT | 2 | GCTGGGACACGAAAGTTACCAA | 22 | 50.00 | 61.25 |
| TBE_LJ_32_RIGHT | 2 | TGCCAAATCTGTCATCCAAGGG | 22 | 50.00 | 61.07 |
| TBE_LJ_33_LEFT | 1 | AATCCGAATGATGGAGGGGGAA | 22 | 50.00 | 61.43 |
| TBE_LJ_33_RIGHT | 1 | CTTTTGAAAGGCAGGCCGTCTC | 22 | 54.55 | 62.45 |
| TBE_LJ_34_LEFT | 2 | CCAAGATGAACTCGTTGGGAGG | 22 | 54.55 | 61.12 |
| TBE_LJ_34_RIGHT | 2 | TCTTGGCCCATTCTGCTCTTTC | 22 | 50.00 | 61.06 |
| TBE_LJ_35_LEFT | 1 | TGTTCCGTACCTCCCTAAAGCT | 22 | 50.00 | 61.01 |
| TBE_LJ_35_RIGHT | 1 | AGCTCAGCCTATTTGCTTTGTCA | 23 | 43.48 | 61.07 |
| TBE_LJ_36_LEFT | 2 | AGCCAGAATTGAGCTGAACCTG | 22 | 50.00 | 61.06 |
| TBE_LJ_36_RIGHT | 2 | TTCGGCCTTATCATGATGCAGG | 22 | 50.00 | 60.99 |
| TBE_LJ_37_LEFT | 1 | CGAAGCCACAGATCATGGAATG | 22 | 50.00 | 59.89 |
| TBE_LJ_37_RIGHT | 1 | GTGGCTCAGGGAGAACAAGAAC | 22 | 54.55 | 61.31 |

Final concentration of each primer in 25 uL PCR reaction is 0.015 uM.
Working concentration of primers is 10 uM - add 0.375 uL oligo into reaction.

| | PCR |
|---|---|
| Pool 1 | 0.715 uL |
| Pool 2 | 0.675 uL |
