# Supplementary material for: Complete Genome Sequencing of Tick-Borne Encephalitis Virus Directly from Clinical Samples: Comparison of Shotgun Metagenomic and Targeted Amplicon-Based Sequencing
Source: Viruses. 2022 Jun 10;14(6):1267. doi: 10.3390/v14061267 (PMC9231111; doi:10.3390/v14061267)
Supplement: Supplementary file 1 [file viruses-14-01267-s001.zip › Supplemental tables/Supplemental Table S3.pdf]

Table S3: Scheme for dividing primer pools into 9 PCR reactions

1	2	3	4	5	6	7	8	9
TBE_LJ_5_LEFT	TBE_LJ_25_LEFT	TBE_LJ_2_LEFT	TBE_LJ_14_LEFT	TBE_LJ_26_LEFT	TBE_LJ_3_LEFT	TBE_LJ_19_LEFT	TBE_LJ_4_LEFT	TBE_LJ_20_LEFT
TBE_LJ_5_RIGHT	TBE_LJ_25_RIGHT	TBE_LJ_2_RIGHT	TBE_LJ_14_RIGHT	TBE_LJ_26_RIGHT	TBE_LJ_3_RIGHT	TBE_LJ_19_RIGHT	TBE_LJ_4_RIGHT	TBE_LJ_20_RIGHT
TBE_LJ_9_LEFT	TBE_LJ_29_LEFT	TBE_LJ_6_LEFT	TBE_LJ_18_LEFT	TBE_LJ_30_LEFT	TBE_LJ_7_LEFT	TBE_LJ_23_LEFT	TBE_LJ_8_LEFT	TBE_LJ_24_LEFT
TBE_LJ_9_RIGHT	TBE_LJ_29_RIGHT	TBE_LJ_6_RIGHT	TBE_LJ_18_RIGHT	TBE_LJ_30_RIGHT	TBE_LJ_7_RIGHT	TBE_LJ_23_RIGHT	TBE_LJ_8_RIGHT	TBE_LJ_24_RIGHT
TBE_LJ_13_LEFT	TBE_LJ_33_LEFT	TBE_LJ_10_LEFT	TBE_LJ_22_LEFT	TBE_LJ_34_LEFT	TBE_LJ_11_LEFT	TBE_LJ_27_LEFT	TBE_LJ_12_LEFT	TBE_LJ_36_LEFT
TBE_LJ_13_RIGHT	TBE_LJ_33_RIGHT	TBE_LJ_10_RIGHT	TBE_LJ_22_RIGHT	TBE_LJ_34_RIGHT	TBE_LJ_11_RIGHT	TBE_LJ_27_RIGHT	TBE_LJ_12_RIGHT	TBE_LJ_36_RIGHT
TBE_LJ_17_LEFT	TBE_LJ_37_LEFT	TBE_LJ_16_LEFT	TBE_LJ_28_LEFT		TBE_LJ_15_LEFT	TBE_LJ_31_LEFT		TBE_LJ_32_LEFT
TBE_LJ_17_RIGHT	TBE_LJ_37_RIGHT	TBE_LJ_16_RIGHT	TBE_LJ_28_RIGHT		TBE_LJ_15_RIGHT	TBE_LJ_31_RIGHT		TBE_LJ_32_RIGHT
TBE_LJ_21_LEFT						TBE_LJ_35_LEFT		
TBE_LJ_21_RIGHT						TBE_LJ_35_RIGHT		
						TBE_LJ_1_LEFT		
						TBE_LJ_1_RIGHT		

All primers in equal volumes and concentrations.
